# Intertissue small RNA communication mediates the acquisition and inheritance of hormesis in *Caenorhabditis elegans*

**DOI:** 10.1038/s42003-021-01692-3

**Published:** 2021-02-16

**Authors:** Emiko Okabe, Masaharu Uno, Saya Kishimoto, Eisuke Nishida

**Affiliations:** 1 RIKEN Center for Biosystems Dynamics Research, 2-2-3 Minatojima Minamimachi, Chuo-ku, Kobe, 650-0047 Japan; 2 Department of Cell and Developmental Biology, Graduate School of Biostudies, Kyoto University, Sakyo-ku, Kyoto, 606-8502 Japan

**Keywords:** Epigenetics, Small RNAs, Epigenetic memory

## Abstract

Environmental conditions can cause phenotypic changes, part of which can be inherited by subsequent generations via soma-to-germline communication. However, the signaling molecules or pathways that mediate intertissue communication remain unclear. Here, we show that intertissue small RNA communication systems play a key role in the acquisition and inheritance of hormesis effects – stress-induced stress resistance – in *Caenorhabditis elegans*. The miRNA-processing enzyme DRSH-1 is involved in both the acquisition and the inheritance of hormesis, whereas worm-specific Argonaute (WAGO) proteins, which function with endo-siRNAs, are involved only in its inheritance. Further analyses demonstrate that the miRNA production system in the neuron and the small RNA transport machinery in the intestine are both essential for its acquisition and that both the transport of small RNAs in the germline and the germline Argonaute HRDE-1 complex are required for its inheritance. Our results thus demonstrate that overlapping and distinct roles of small RNA systems in the acquisition and inheritance of hormesis effects.

## Abstract (web summary)

Okabe et al. show that the miRNA production system in the neuron and the small RNA transport machinery in the intestine are required for the acquisition of hormesis. For its inheritance, both the transport of small RNAs in the germline and the germline Argonaute HRDE-1 complex are needed, highlighting distinct contribution of small RNA systems to hormesis.

## Introduction

Organisms are constantly exposed to various types of environmental stresses. These stresses can affect organismal phenotypes through gene expression alterations, and these acquired phenotypes are sometimes inherited to future generations. In *Caenorhabditis elegans*, several studies have shown that various environmental stimuli (e.g., viral infection, starvation, high-fat diet, and heat stress) induce heritable effects^1–4^. Recently, we have shown that exposure to various mild stresses during developmental stages results in the increase in oxidative stress resistance of animals in a process known as hormesis, and this hormesis effect can be transmitted to subsequent generations via soma-to-germline communication^5^. Hormesis is a biological phenomenon whereby exposure to low levels of toxic agents or conditions increases organismal viability. Transmission of a heritable trait over generations requires accurate intergenerational regulation of several sets of genes responsible for the trait in descendants. However, it remains unknown how parental generations transform environmental information into epigenetic changes in specific genes during soma-to-germline communication.

Small RNAs are one of the key players in epigenetic mechanisms, such as histone modifications and DNA methylation, and play an essential role in various biological processes. Small RNAs are grouped into three classes: PIWI-interacting RNA (piRNAs), micro RNAs (miRNAs), and endogenous small-interfering RNAs (endo-siRNAs). These small RNAs need to be loaded onto Argonaute (AGO) proteins to form the effector complex as an RNA-induced silencing complex (RISC). RISC transcriptionally and post-transcriptionally silences target genes, in which the sequence specificity in silencing is rendered by small RNAs^6^. Thus, these small RNAs are plausible candidates for the intergenerational regulation of specific genes. Although several studies have demonstrated that small RNAs can mediate transgenerational epigenetic inheritance^7–9^, the role of small RNAs in the transgenerational inheritance of hormesis effects has not been explored.

In this study, we examined the role of small RNAs in the acquisition and inheritance of hormesis, that is, the osmotic stress-induced increase in resistance to oxidative stress in *C. elegans*. Our results show that the miRNA-processing enzyme Drosha/DRSH-1 is required for both the acquisition of hormesis and its transmission to the next generation. In contrast, worm-specific Argonaute (WAGO) proteins, which function in the endo-siRNA pathway, are required only for its inheritance. Our analyses demonstrate that the germline nuclear Argonaute complex transmits hormesis to the offspring through the putative H3K9 methyltransferases SET-25 and SET-32. Moreover, we found that the miRNA production in the parental neuronal tissues is essential for the transmission of hormesis via the small RNA channel SID-1 in the intestine and germline. Our results thus reveal that intertissue communications by two distinct classes of small RNAs mediate the conversion of parental environmental conditions into epigenetic information, which could be maintained and inherited to the offspring.

## Results

### MiRNAs and endo-siRNAs differentially function in the inheritance of hormesis effect

**Fig. 1:**
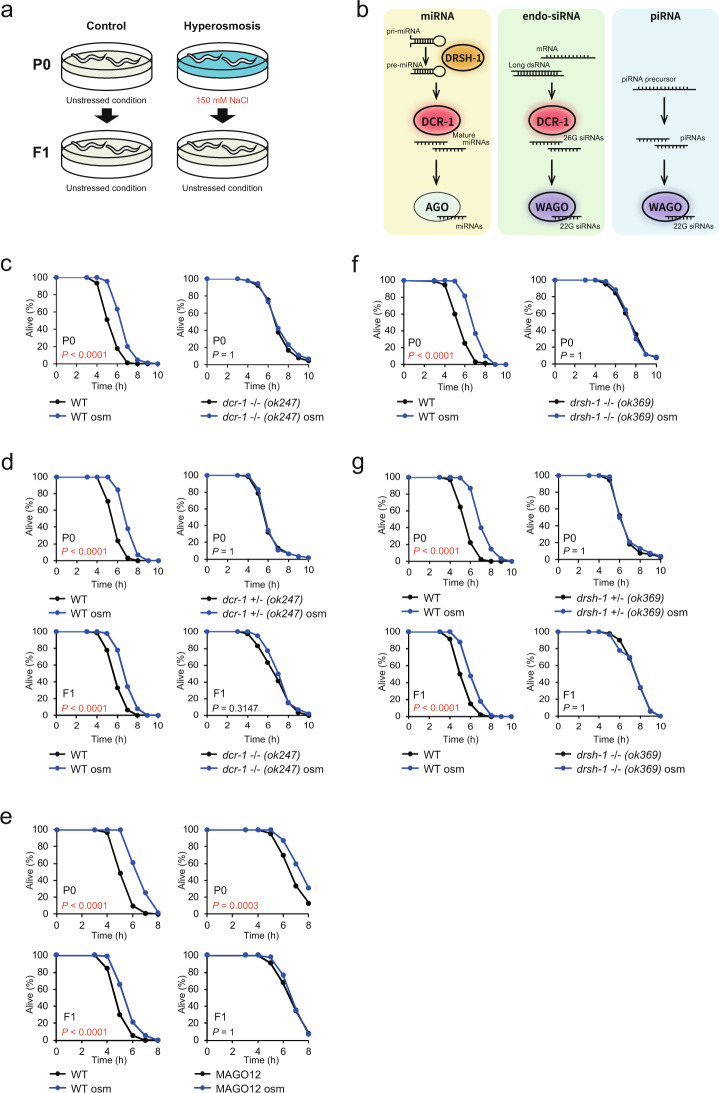
MiRNAs and endo-siRNAs differentially mediate the acquisition and inheritance of hormesis. **a** Scheme for exposure to the environmental stress condition. The P0 parents were exposed to hyperosmosis during developmental stages and the F1 descendants were raised under unstressed conditions. **b** Overview of small RNA pathways in *C. elegans*. **c** Oxidative stress resistance (2.0 mM H_2_O_2_) of wild-type (WT) N2 worms (left) and *dcr-1* homozygotes (right) in the P0 generation. **d** Oxidative stress resistance of WT (upper left) and *dcr-1* heterozygotes (upper right) in the P0 generation and WT (lower left) and *dcr-1* homozygotes (lower right) in the F1 generation. **e** Oxidative stress resistance (1.8 mM H_2_O_2_) of WT (left) and MAGO12 mutants (right) in the P0 generation (upper) and the F1 generation (lower). **f** Oxidative stress resistance (2.0 mM H_2_O_2_) of WT (left) and *drsh-1* homozygotes (right) in the P0 generation. **g** Oxidative stress resistance of WT (upper left) and *drsh-1* heterozygotes (upper right) in the P0 generation and WT (lower left) and *dcr-1* homozygotes (lower right) in the F1 generation. Homozygotes of *dcr-1* or *drsh-1* were distinguished from heterozygotes by GFP expression. Three independent experiments are integrated into each survival curve (*n* = 90). Mean survival time and statistics are presented in Supplementary Data 1. *P* values were calculated by log-rank test with Bonferroni correction.

As soma-to-germline communication is important for the acquisition and transmission of hormesis^5^, we examined the role of small RNAs, which function in somatic tissues: endo-siRNAs and miRNAs, in these processes (Fig. 1a). We first focused on the RNase Dicer/DCR-1, which is essential for the generation of both endo-siRNAs and miRNAs (Fig. 1b)^10,11^. We raised worms (the P0 parents) on high-salt media plates (hyperosmosis conditions) during larval stages and their offspring (the F1 descendants) on normal salt media plates (unstressed conditions) (Fig. 1a). Then, we measured the stress resistance of these P0 and F1 animals to a fatal oxidative stressor, hydrogen peroxide (H_2_O_2_). While the exposure to hyperosmosis during larval stages increased the oxidative stress resistance of wild-type animals as reported^5^, it did not increase that of the *dcr-1(ok247)* homozygous animals (Fig. 1c). We could not analyze the stress resistance of the F1 descendants of the *dcr-1(ok247)* homozygous because *dcr-1(ok247)* homozygous showed sterility. To obtain the F1 *dcr-1* homozygous mutants, we raised parental *dcr-1(ok247)* heterozygotes under hyperosmosis and used these heterozygotes as P0 parents and their offspring homozygotes, which were raised under unstressed conditions, as F1 descendants. Wild-type animals showed an increase in stress resistance in both the P0 parents and the F1 descendants (Fig. 1d) as reported^5^. On the other hand, *dcr-1(ok247)* heterozygotes (the P0 parents) and homozygotes (the F1 descendants) did not show increased stress resistance (Fig. 1d). Moreover, we analyzed another *dcr-1* mutant *(tm12491)*, which is fertile. The *dcr-1 (tm12491)* mutant also did not display increased stress resistance in both the P0 and F1 generations (Supplementary Fig. 1a). The unstressed *dcr-1(ok247)* homozygotes displayed increased stress resistance compared with unstressed WT animals, but the unstressed *dcr-1(tm12491)* homozygotes did not. This may result from the difference in fertility, because sterility increases lifespan and stress resistance to pathogen infection^12,13^. Consistently, the *dcr-1(ok247)* heterozygotes did not display increased stress resistance (Fig. 1d) and knockdown of *dcr-1*, after completion of development, did not increase stress resistance (Supplementary Fig. 1b–d). Remarkably, the *dcr-1(ok247)* heterozygous mutant did not display the hyperosmosis-induced increase in stress resistance in the P0 generation (Fig. 1d), and knockdown of *dcr-1* after completion of development did not increase stress resistance in the P0 generation (Supplementary Fig. 1c). These data imply that DCR-1, but not sterility, is involved in hyperosmosis-induced hormesis. Thus, these results suggest that the production of endo-siRNAs and/or miRNAs is required for the acquisition and possibly the transmission of the increased oxidative stress resistance.

To analyze the role of endo-siRNAs and miRNAs in more detail, we focused on the WAGOs. *C. elegans* has 27 AGO proteins, 12 of which are WAGO proteins, which function with a class of endo-siRNAs (called 22G siRNAs) to silence target genes (Fig. 1b)^11,14,15^. We utilized the MAGO12 mutant, which lacks all 12 WAGO encoding genes and is deficient in RNA interference (RNAi) induced by endo-siRNAs^16^. In MAGO12 mutants, hyperosmosis exposure during larval stages increased the stress resistance of the P0 animals but not that of the F1 animals (Fig. 1e), suggesting that endo-siRNAs have a role not in the acquisition of hormesis but in its transmission.

Because the acquisition of hormesis requires DCR-1, which is involved in both endo-siRNA and miRNA functions, but it does not require WAGOs, which are involved only in endo-siRNA function, miRNAs may have a role in the acquisition. To test this possibility, we used the mutants of *drsh-1*, the gene encoding the miRNA-processing enzyme Drosha/DRSH-1, which executes the initial step of miRNA processing (Fig. 1b). Hyperosmosis exposure did not increase the oxidative stress resistance of *drsh-1* homozygous animals in the P0 generation (Fig. 1f). Because *drsh-1* homozygous animals, such as *dcr-1* homozygous animals, also exhibited sterility, we measured the stress resistance of heterozygotes in the P0 parents and their homozygous progeny in the F1 descendants. Both the *drsh-1* heterozygotes in the P0 generation and the homozygotes in the F1 generation did not show increased stress resistance (Fig. 1g). Moreover, we knocked down *drsh-1* after completion of development. The *drsh-1*-knockdown animals, whose basal stress resistance was comparable to that of wild-type, did not display the increased stress resistance in the P0 and F1 generations (Supplementary Fig. 1b–d). These results indicate that DRSH-1 is required for the acquisition of hormesis and possibly for its inheritance. Thus, these findings suggest that miRNAs play an essential role in both the acquisition and the inheritance of hormesis, whereas endo-siRNAs play a role only in the inheritance.

To further examine the role of *dcr-1* and *drsh-1* in the stress resistance of the unstressed F1 generations, we knocked down *dcr-1* or *drsh-1* only in the F1 generation, whose parents were exposed to hyperosmosis during larval stages. Neither *dcr-1*-knockdown F1 animals nor *drsh-1*-knockdown F1 animals showed increased stress resistance (Supplementary Fig. 1e, 1f), suggesting that the increased stress resistance in the F1 generation requires both endo-siRNAs and miRNAs. Therefore, both endo-siRNAs and miRNAs in the F1 generation are essential for the intergenerational inheritance of hormesis.

### Germline nuclear RNAi pathway is required for the inheritance of hormesis

**Fig. 2:**
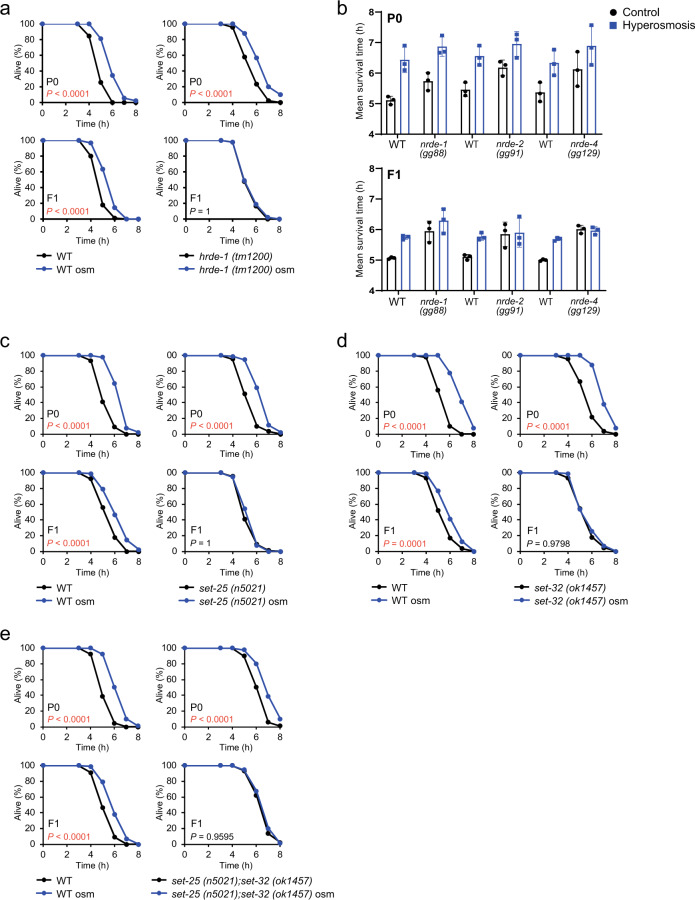
Components in the germline nuclear RNAi pathway are required for the inheritance of hormesis effect. **a** Oxidative stress resistance (1.8 mM H_2_O_2_) of WT (left) and *hrde-1* mutants (right) in the P0 generation (upper) and the F1 generation (lower). **b** The mean survival time to oxidative stress of three independent experiments is shown. Error bars, S.D. **c** Oxidative stress resistance of WT (left) and *set-25* mutants (right) in the P0 generation (upper) and the F1 generation (lower). **d** Oxidative stress resistance of WT (left) and *set-32* mutants (right) in the P0 generation (upper) and the F1 generation (lower). **e** Oxidative stress resistance of WT (left) and *set-25;set-32* double-mutants (right) in the P0 generation (upper) and the F1 generation (lower). Three independent experiments are integrated into each survival curve (*n* = 90). Mean survival time and statistics are presented in Supplementary Data 1. *P* values were calculated by log-rank test with Bonferroni correction.

Endo-siRNAs are required for the inheritance of hormesis but not for its acquisition. This raises the possibility that endo-siRNAs in the germline might play a critical role in the transmission of hormesis to the next generation. To test this possibility, we examined the role of HRDE-1, which is the germline nuclear WAGO protein^17^, in the acquisition and inheritance of hormesis. The *hrde-1* mutant animals showed increased oxidative stress resistance in the P0 generation but not in the F1 descendants (Fig. 2a). Moreover, we knocked down *hrde-1* after completion of development. The *hrde-1*-knockdown animals, whose basal stress resistance was comparable to that of wild-type, displayed the increased stress resistance in the P0 generation but not in the F1 generation (Supplementary Fig. 2). Because HRDE-1 functions with NRDE factors (NRDE-1, -2, and -4) to transcriptionally suppress target genes^8,17–19^, we examined the role of NRDE factors. Our analysis showed that hyperosmosis exposure increased the oxidative stress resistance in the P0 generation of *nrde-1, -2, and -4* mutant animals but not in their F1 descendants (Fig. 2b). These results indicate that the germline nuclear Argonaute protein HRDE-1 and its partners are required for the inheritance of hormesis.

HRDE-1 with endo-siRNAs silences gene expression in the nucleus^17,20,21^. Thus, HRDE-1 and its partners (NRDE factors) inhibit RNA polymerase II and promote methylation of histone H3 lysine 9 (H3K9) in the germline nuclear RNAi pathway^8,17,20,22^. This H3K9 methylation requires putative H3K9 methyltransferases SET-25 and SET-32^23–25^. Next, we examined whether SET-25 and SET-32 are necessary for the inheritance of hormesis. The results demonstrated that *set-25* or *set-32* mutants showed increased stress resistance in the P0 generation, but their F1 descendants did not (Figs. 2c, 2d). We also examined *set-25;set-32* double-mutant animals and found that the double-mutant animals showed increased stress resistance in the P0 generation, but their F1 progeny did not (Fig. 2e). These results demonstrate that epigenetic alterations caused by SET-25 and SET-32 may play an important role in the inheritance of hormesis effect and imply that the intergenerational inheritance of hormesis effect should be mediated through epigenetic modifications following HRDE-1 complex recruitment to the target sites via endo-siRNAs.

### Intertissue transport of small RNAs is required for both the acquisition and the inheritance of hormesis

**Fig. 3:**
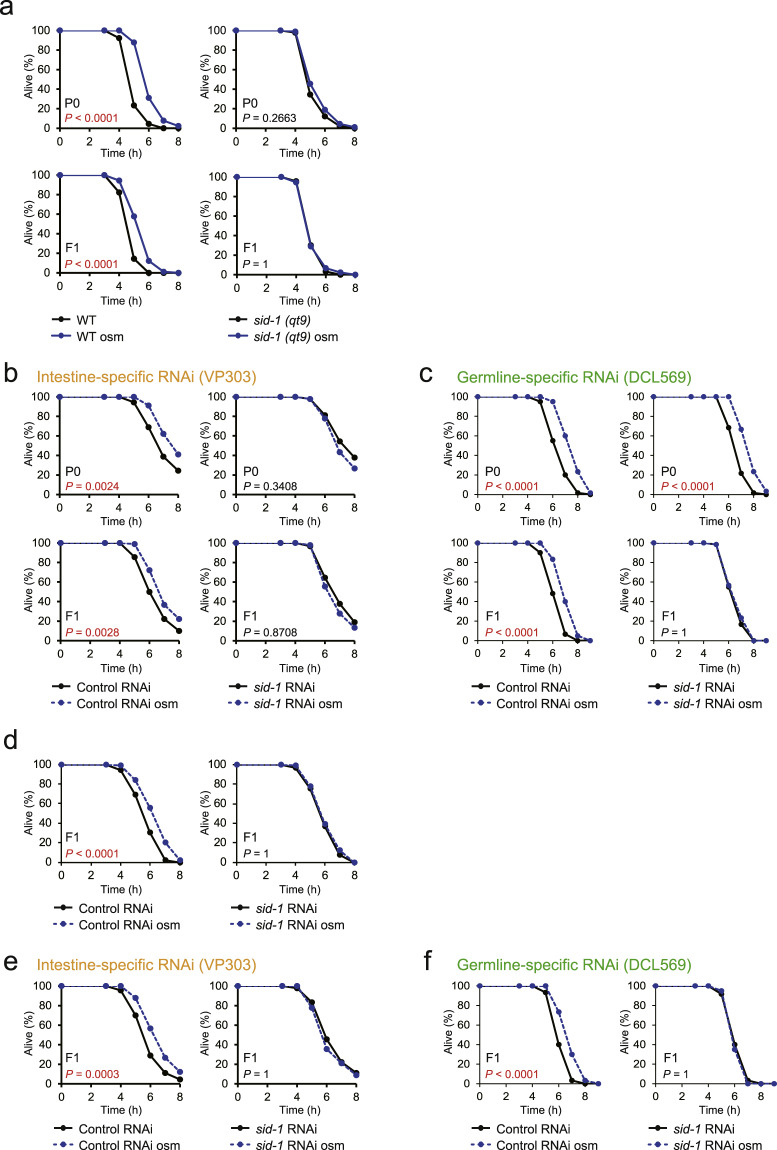
Intertissue transport of small RNAs is necessary for both the acquisition and the inheritance of hormesis. **a** Oxidative stress resistance (1.8 mM H_2_O_2_) of WT (left) and *sid-1* mutants (right) in the P0 generation (upper) and the F1 generation (lower). **b** Oxidative stress resistance (2.5 mM H_2_O_2_) of intestine-specific RNAi mutants (VP303) treated with RNAi (left, control RNAi; right, *sid-1* RNAi) in the P0 generation (upper) and the F1 generation (lower). **c** Oxidative stress resistance (2.0 mM H_2_O_2_) of germline-specific RNAi mutants (DCL569) treated with RNAi (left, control RNAi; right, *sid-1* RNAi) in the P0 generation (upper) and the F1 generation (lower). **d** Oxidative stress resistance (2.0 mM H_2_O_2_) of the F1 descendants, which were derived from the stressed P0 parents and treated with RNAi (left, control RNAi; right, *sid-1* RNAi). **e** Oxidative stress resistance (2.5 mM H_2_O_2_) of the F1 descendants in intestine-specific RNAi mutants, which were derived from the stressed P0 parents and treated with RNAi (left, control RNAi; right, *sid-1* RNAi). **f** Oxidative stress resistance (2.0 mM H_2_O_2_) of the F1 descendants in germline-specific RNAi mutants, which were derived from the stressed P0 parents and treated with RNAi (left, control RNAi; right, *sid-1* RNAi). Two or three independent experiments are integrated into each survival curve (*n* = 60 or 90). Mean survival time and statistics are presented in Supplementary Data 1. *P* values were calculated by log-rank test with Bonferroni correction.

We hypothesized that small RNAs transduce the environmental information across tissues and that the transported small RNAs in the germline play an essential role in the phenotypes of the offspring. The hypothesis could be derived from the results described above together with the following observations: (1) our previous study revealed that germline-to-soma communication has an important role in the transgenerational hormesis effect^5^. (2) Exogenous dsRNA introduction, which induces the production of exogenous siRNAs (exo-siRNAs), in one tissue elicits silencing of target genes not only in that tissue but also in other tissues^26^. (3) Exo-siRNAs and endo-siRNAs share the mechanisms of silencing target genes^11,27^. Because the transport of dsRNA into cells was shown to require a dsRNA channel SID-1^28^, we analysed the effect of *sid-1* mutation on hormesis and its inheritance. We found that the increased oxidative stress resistance by hyperosmosis was suppressed in both the P0 and F1 generations of *sid-1* mutants (Fig. 3a), suggesting that the intercellular transport of small RNAs is essential for the acquisition and possibly the transmission of stress-induced stress resistance.

To determine tissues to which small RNAs are transmitted, we performed tissue-specific RNAi experiments using strains that are able to process RNAi efficiently only in particular tissues, such as intestine and neuron, but not in other tissue (intestine: VP303^29^; germline: DCL569^30^). Treatment with intestine-specific *sid-1* RNAi suppressed the increase in stress resistance completely in the P0 generations (Fig. 3b, upper). On the other hand, treatment with germline-specific *sid-1* RNAi did not suppress the increase in stress resistance in the P0 generations (Fig. 3c, upper). Both the intestine-specific and germline-specific *sid-1* RNAi treatments completely suppressed the stress resistance increase in the descendants (Figs. 3b, 3c, lower). These data suggest that the acquisition of hormesis depends on SID-1 function in the intestine and that the inheritance of this hormesis effect requires the transmission of small RNAs to the germline in the P0 generations. This is consistent with our idea that the germline nuclear RNAi pathway in the P0 generation is essential for the inheritance of hormesis.

To further examine whether the intertissue transport of small RNAs is required to ensure the increased stress resistance in F1 descendants, we knocked down *sid-1* only in the F1 generation. The entire body knockdown of *sid-1* in the F1 generation suppressed the increase in the stress resistance (Fig. 3d, Supplementary Fig. 3). Then, we performed tissue-specific *sid-1* RNAi. Knockdown of *sid-1* in the intestine or germline in the F1 generation led to the suppression of the increase in stress resistance (Figs. 3e, 3f), demonstrating that transport of small RNAs to the intestine and germline is required to increase the stress resistance in the F1 descendants. Taken together, these findings suggest that the intertissue transport of small RNAs between soma and germ cells is required for not only the acquisition of hormesis in the P0 generation but also its inheritance in the F1 generation.

### Production of miRNAs in the neuron and intestine is required for the inheritance of hormesis

Our results suggest that the intergenerational hormesis effect requires intertissue small RNA communication. Then, we explored the tissues in which DCR-1 and DRSH-1, the enzymes essential for producing small RNAs, play a role. To this end, we performed knockout experiments. According to the method used in a previous study^31^, we knocked out *dcr-1* or *drsh-1* in the somatic tissue, neuron, or intestine by using CRISPR/Cas9 system with the *eft-3*, *rgef-1* or *gly-19* promoter, respectively. The animals whose *dcr-1* or *drsh-1* in somatic tissues, neuron, and intestine was knocked out were apparently healthy and fertile. To visualize the tissues in which *dcr-1* or *drsh-1*-knockout was performed, we introduced sgRNA and Cas9 with a *sur-5* promoter (all somatic tissues) driven by wGxxFP fused to the nuclear localization signal (NLS::wGxxFP), which is reconstituted into GFP after homology-dependent repair (HDR) caused by sgRNA-guided Cas9-induced digestion^32^. When we introduced Cas9 into the neuron and intestine, we detected GFP fluorescence in the neuronal and intestinal nuclei, respectively. (Supplementary Fig. 4a–d), suggesting that our tissue-specific knockout experiment worked properly.

Knockout of *dcr-1* in somatic tissues suppressed the increase in the oxidative stress resistance in the P0 generations (Supplementary Fig. 4e, upper). Knockout of *drsh-1* in somatic tissues moderately suppressed the increase in the stress resistance in the P0 generation, although the difference in the stress resistance between unstressed and stressed *drsh-1*-knockout animals was statistically significant (*p* = 0.008, Supplementary Fig. 4f, upper). Either knockout of *dcr-1* and *drsh-1* in somatic tissues suppressed the increase in the stress resistance in F1 generations (Supplementary Fig. 4e and 4f, lower). These results suggest that the small RNA production in somatic tissues contributes to the acquisition of hormesis and possibly its inheritance.

**Fig. 4:**
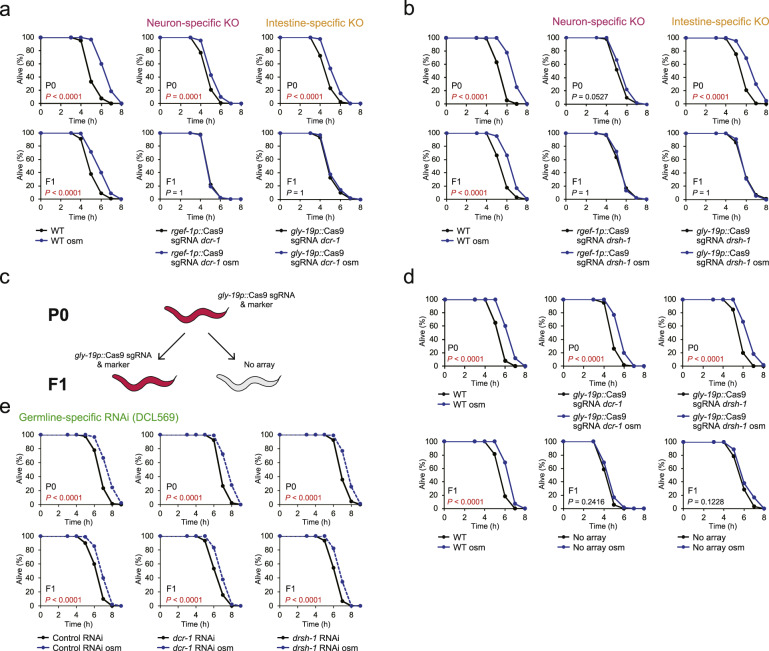
Production of miRNAs in the neuron and intestine is necessary for the inheritance of hormesis. **a** Oxidative stress resistance (1.8 mM H_2_O_2_) of WT (left), neuron-specific *dcr-1*-knockout mutants (middle) and intestine-specific *dcr-1*-knockout mutants (right) in the P0 generation (upper) and the F1 generation (lower). **b** Oxidative stress resistance of WT (left), neuron-specific *drsh-1*-knockout mutants (middle), and intestine-specific *drsh-1*-knockout mutants (right) in the P0 generation (upper) and the F1 generation (lower). **c** Scheme for obtaining the descendants (no array) from intestine-specific knockout worms. The descendants (no array) were distinguished by the loss of a mCherry fluorescent coinjection marker. **d** Oxidative stress resistance of WT (left), intestine-specific *dcr-1*-knockout mutants (middle), and intestine-specific *drsh-1*-knockout mutants (right) in the P0 generation (upper) and in the F1 generation (no array) (lower). **e** Oxidative stress resistance (2.0 mM H_2_O_2_) of germline-specific RNAi mutants (DCL569) treated with RNAi (left, control RNAi; middle. *dcr-1* RNAi; right, *drsh-1* RNAi) in the P0 generation (upper) and the F1 generation (Lower). Two or three independent experiments are distinguished into each survival curve (*n* = 60 or 90). Mean survival time and statistics are presented in Supplementary Data 1. *P* values were calculated by log-rank test with Bonferroni correction.

Because hyperosmosis in the environment is sensed by chemosensory neurons, which are required for osmotic avoidance^33^, we examined whether the function of DCR-1 and DRSH-1 in the neuron is required for the intergenerational hormesis effect. To this end, we performed neuron-specific *dcr-1* or *drsh-1*-knockout. Knockout of *dcr-1* and *drsh-1* in the neuron partially and completely suppressed the increase in oxidative stress resistance of the P0 parents and F1 descendants, respectively (Figs. 4a, 4b, middle). These results indicate that the small RNA production in the neuron plays an important role in both the acquisition and the inheritance of the stress-induced hormesis effect.

Then, we examined the role of DCR-1 or DRSH-1 in the intestine in the inheritance of hormesis. As the excretory/secretory function is adopted in the intestine in *C. elegans*, the intestine should have an important role in the osmotic stress response. The extent of the stress resistance that increases in the intestine-specific *dcr-1* or *drsh-1*-knockout animals was smaller than that in wild-type animals in the P0 parents (Figs. 4a, 4b, upper right), suggesting that the small RNA production in the intestine might be partially involved in acquisition of hormesis. On the other hand, the intestine-specific *dcr-1* or *drsh-1*-knockout completely suppressed the stress resistance increase in the F1 descendants (Figs. 4a, 4b, lower right). The suppression of the heritable hormesis effect could be due to the inhibition of small RNA production in the P0 and/or F1 generations. We then asked whether the intestine-specific *dcr-1* or *drsh-1*-knockout in the P0 generation has a role in the heritable hormesis effect in the F1 descendants. In *C. elegans*, the injected exogenous DNA can be propagated as extrachromosomal arrays that contain multiple copies of the injected DNA^34^, and the extrachromosomal arrays can be randomly lost during mitotic division^35^. Thus, by using the intestine-specific *dcr-1* or *drsh-1*-knockout transgenic animals as the P0 parents, we can obtain the F1 descendants without extrachromosomal arrays (hereafter named “no array”), which are genetically identical to wild-type animals (Fig. 4c). When intestine-specific *dcr-1* or *drsh-1*-knockout parents showed an increase in oxidative stress resistance, the descendants (no array) did not show increased stress resistance (Fig. 4d). This result suggests that the inheritance of hormesis to descendants requires DCR-1 and DRSH-1 in the intestine of the P0 parents.

Because the germline is an important tissue for the transmission of parental information across generations, we examined whether the function of DCR-1 and DRSH-1 in the germline is required for the intergenerational hormesis effect using the germline-specific RNAi method. Neither *dcr-1* RNAi nor *drsh-1* RNAi suppressed the increase in stress resistance in both the P0 and the F1 generations (Fig. 4e). This result suggests that the small RNA production in the germline is not required for the acquisition and inheritance of hormesis. Altogether, our findings suggest that small RNAs, especially miRNAs, in the neuron in animals subjected to hyperosmosis, play an important role in the acquisition of hormesis and that the production of small RNAs in the intestine plays a role in the inheritance of hormesis.

## Discussion

Our previous study demonstrated that a stress-induced hormesis effect can be transmitted over generations through soma-to-germline communications in *C. elegans*^5^. However, the molecules responsible for intertissue communication remain unknown. In this study, we have shown the essential role of the intertissue small RNA communication systems in the inheritance of hormesis in *C. elegans*. Our results demonstrate that the germline nuclear RNAi pathway functions in the inheritance of hormesis, consistent with previous studies showing that RNAi mediates transgenerational gene silencing through the germline nuclear RNAi pathway in *C. elegans*^17,36^. Moreover, our results also show that miRNAs, in addition to endo-siRNAs, function in the acquisition and inheritance of hormesis. As several miRNAs have been shown to be involved in stress responses^37^, it is reasonable that miRNAs, as well as endo-siRNAs, function in the intergenerational hormesis effect caused by environmental perturbation. Our analyses suggest that miRNA production, but not endo-siRNA function, is required for the acquisition of hormesis and that miRNA and endo-siRNA productions are both required for the inheritance of hormesis to the offspring. Our data further suggest that miRNA production in the intestine, but not in the germline, is involved in the inheritance of hormesis, and endo-siRNA function in the germline and possibly that in the intestine are involved in the inheritance of hormesis. Since we examined hermaphrodite animals under test conditions in this study, it cannot be denied that maternal small RNAs might be directly transmitted from the intestine to the embryos.　However, as our results have shown that the germline Argonaute HRDE-1 is required for the inheritance of hormesis, it is plausible that the intergenerational inheritance of hormesis could be regulated by the intestine-to-germline small RNA signaling. This idea is consistent with our previous observation that the F1 cross-progeny whose parental males were exposed to the hyperosmosis stress showed increased stress resistance^5^.

Because the intestine miRNA production and the germline endo-siRNA function are important for the inheritance of hormesis effects, the hyperosmosis-induced miRNA production in the intestine could modulate the endo-siRNA function in the germline. These findings raise the possibility that cross-talk exists between miRNA and endo-siRNA-mediated pathways; worm might directly or indirectly convert miRNAs to endo-siRNAs to transmit the environmental information to the descendants. The specific miRNA has been shown to regulate the production of endo-siRNA^38^, in agreement with our hypothesis. However, it remains unclear where and how miRNAs affect endo-siRNA. Further studies will be needed to determine how cross-talk between these small RNAs in the parental generation contributes to the inheritance of the acquired traits over generations.

Based on our results, a simplified working hypothesis may be proposed, in which the inheritance of hormesis may occur in three steps: acquisition, transmission, and maintenance (Supplementary Fig. 5). First, the acquisition of hormesis requires DCR-1 and DRSH-1 in the neuron and SID-1 in the intestine, suggesting the possibility that miRNAs, which are produced in response to environmental changes, move from the neuron to the intestine, and these transported miRNAs may contribute to an increase in organismal stress resistance. This is because several studies have shown that neurotransmitters regulate the function in distal tissues^39,40^, and that the intestine plays a central role in the adaptation to hyperosmosis^41,42^ and the response to the oxidative stress^43^. Second, the transmission of hormesis requires DRSH-1 and DCR-1 in the intestine, the components of the germline nuclear RNAi pathway, and SID-1 in the germline. These results imply that small RNAs produced in the intestine may translocate to the germline and function there through histone modification via the germline nuclear RNAi pathway. Consistently, the interactions between the intestine and the germline have been reported; the intestine interacts with the germline to regulate lipid metabolism and lifespan^44,45^, and the intestine-to-germline communication intergenerationally regulates stress resistance through histone modifications^46^. Finally, the maintenance of the epigenetic alterations in the descendants requires DRSH-1, DCR-1, and SID-1 in the descendant, suggesting that small RNA production and intertissue communication may ensure increased stress resistance in the offspring. Thus, the present study deepens our understanding of intertissue communication through small RNAs, which contributes to the transmission of environmental information to descendants as epigenetic alterations.

In summary, our results show that the miRNA production system is required for both the acquisition and the inheritance of hormesis, whereas endo-siRNA function is required only for its inheritance, and that the intertissue communication of these small RNAs is indispensable for both the acquisition and the inheritance of hormesis. Thus, this study uncovers a potential strategy in which the conversion of parental experiences to epigenetic information contributes to intergenerational inheritance of the acquired traits.

Most recently, two papers reported the importance of endo-siRNAs in the transgenerational inheritance of acquired traits. One shows that the endo-siRNA regulators are required for transgenerational learned pathogen avoidance^47^, and the other shows that neuronal endo-siRNAs communicate with the germline to control behavior transgenerationally^48^. These studies, together with our study, reveal the essential role of small RNAs and their intertissue communication in the acquisition and inheritance of phenotypic changes in response to environmental changes.

## Methods

### *C. elegans* strains

All nematodes were cultured using standard *C. elegans* methods^49^. All experiments were performed at 20 °C. The strains are listed in Supplementary Table 1.

### Environmental stress conditions

Synchronized eggs were obtained by the bleaching method^50^. Using these synchronous eggs, animals were raised on high-salt growth media plates (150 mM NaCl) for 4 days (4 days old animals were defined as day2 adulthood). To obtain animals of the F1 generation, 10–20 gravid day-2 adults, which were stressed or unstressed, were transferred onto new NGM plates without high salt, and the animals were allowed to lay eggs for several hours. The parents were then removed, and the plates were incubated at 20 °C for 4 days.

### Oxidative stress assays

Day2 adults were transferred into each well (60-well plate, Greiner bio-one) containing 20 μl of the M9 buffer containing pro-oxidant (1.8, 2.0, or 2.5 mM hydrogen peroxide) (Santoku Chemical Industries Co., Ltd). The plates were monitored almost every hour to document the number of animals alive or dead. Animals were scored as dead if they failed to respond to touch with a picker. Summaries of stress resistance experiments are presented in Supplementary Data 1.

### RNA interference

RNA interference was performed by the feeding method as described^51^. *Sid-1*, *dcr-1,* and *drsh-1* RNAi clones were obtained from the *C. elegans* RNAi library (Source BioScience). The *hrde-1* RNAi clone was constructed by PCR. The primers used are listed in Supplementary Table 2.

### Quantitative RT-PCR

Total RNA was isolated using TRIzol reagent (Invitrogen) from frozen day2 adults under the indicated RNAi conditions. The extracted total RNA was reverse-transcribed into single-stranded cDNA using the ReverTra Ace qPCR RT Master Mix with gDNA Remover kit (TOYOBO) according to the manufacturer’s protocol. Quantitative RT-PCR was performed with a QuantStudio®3 Real-Time PCR system (Applied Biosystems) using TB Green® Premix Ex Taq^TM^ II (Tli RNase H Plus) (TAKARA). The relative mRNA levels were determined using the standard curve method or the ΔΔCT method and normalized to that of *act-1*, a *C. elegans* housekeeping gene. The primers used are listed in Supplementary Table 2.

### Tissue-specific knockout experiments

Tissue-specific knockouts were performed following the protocol as described^31^. The CRISPR-Cas9 vector was obtained from Addgene (#47549). To express Cas9 in a tissue-specific manner, we cloned promoters (*rgef-1* or *gly-19* promoters for neuron- or intestine-specific expression, respectively). We used the CRISPR design tool (http://crispr.egu) to select *dcr-1* and *drsh-1* sgRNA. To visualize the tissues generated with CRISPR-Cas9, we used the EGxxFP system in *C. elegans*^32^. We modified pPD95.75 to generate the worm GxxFP fused to the nuclear localization signal (NLS::wGxxFP). We inserted the *dcr-*1 or *drsh-1* sgRNA target site in wGxxFP. The expression of the wGxxFP construct was driven by the *sur-5* promoter (in all somatic tissues). The Primers used for *dcr-1* and *drsh-1* sgRNA, and wGxxFP are listed in Supplementary Table 2.

### Fluorescent microscopy

Animals were anesthetized with 2 mM levamisole in M9 buffer on the coverslip with 2% agarose pad and observed with a stereomicroscope SZX16 (Olympus).

### Statistics and reproducibility

Statistical analysis, the number of animals, and the number of replicates for each experiment are indicated in figure legends and Supplementary Data 1. For the oxidative stress assays, *P* values were calculated by log-rank test with Bonferroni correction for multiple comparisons using the Online Application for Survival Analysis 2 (OASIS 2: http://sbi.postech.ac.kr/oasis2)^52^. For the quantitative RT-PCR assays, *P* values were calculated by unpaired Student’s *t*-test using Excel software. Statistical significance was defined as *p* < 0.05.

### Reporting summary

Further information on research design is available in the Nature Research Reporting Summary linked to this article.

## Supplementary information

Supplementary Information

Description of Additional Supplementary Files

Supplementary Data 1

Reporting Summary

## Data Availability

The authors declare that all data supporting the findings of this study are available within the paper and its Supplementary Information Files or from the corresponding author on reasonable request. Source data for the main and supplementary figures are available in Supplementary Data 1.
