# Supplementary material for: Intertissue small RNA communication mediates the acquisition and inheritance of hormesis in Caenorhabditis elegans
Source: Commun Biol. 2021 Feb 16;4:207. doi: 10.1038/s42003-021-01692-3 (PMC7886853; doi:10.1038/s42003-021-01692-3)
Supplement: Supplementary file 1 — Supplementary Information [file 42003_2021_1692_MOESM1_ESM.pdf]

Supplementary Information

Intertissue small RNA communication mediates the acquisition and inheritance of hormesis in *C. elegans*

Emiko Okabe, Masaharu Uno, Saya Kishimoto and Eisuke Nishida

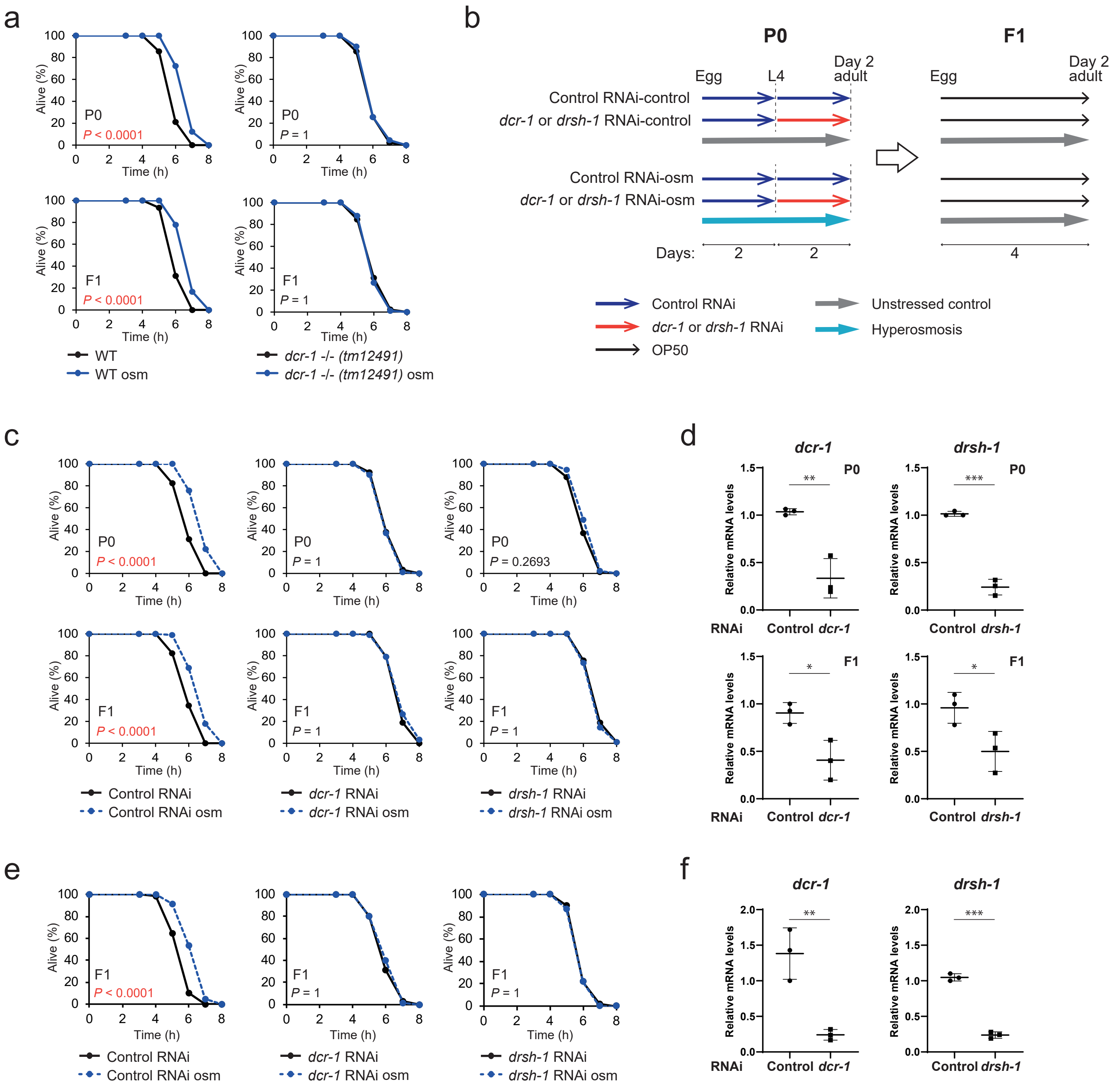

Supplementary Figure 1. Effects of RNAi treatment of *dcr-1* or *drsh-1* on the stress resistance.

a, Oxidative stress resistance (1.8 mM H_2O_2) of WT (left) and *dcr-1* mutants (right) in the P0 generation (upper) and the F1 generation (lower). **b**, Scheme for exposure to the hyperosmotic stress condition and treatment with RNAi. The P0 parents were exposed to hyperosmosis for 4 days from egg stage to day 2 adulthood and treated with RNAi for 2 days from L4 stage to day 2 adulthood. The F1 descendants were raised under unstressed conditions. **c**, Oxidative stress resistance (2.0 mM H_2O_2) of worms treated with RNAi (left, control RNAi; middle, *dcr-1* RNAi; right, *drsh-1*RNAi) in the P0 generation (upper) and the F1 generation (lower). **d**, Relative mRNA expression levels of *dcr-1* and *drsh-1* in the P0 generation (upper) and the F1 generation (lower). Error bars represent S.D. of three independent biological replicates. **e**, Oxidative stress resistance (2.0 mM H_2O_2) of the F1 descendants, which were derived from the stressed P0 parents and treated with RNAi (left, control RNAi; middle, *dcr-1* RNAi; right, *drsh-1* RNAi). **f**, Relative mRNA expression levels of *dcr-1* and *drsh-1* in WT animals. Error bars represent S.D. of three independent biological replicates. *P* values were calculated by log-rank test with Bonferroni correction **a**, **c**, **e** and unpaired Student' s *t*-test (****p*<0.001, ***p*<0.01, **p*<0.05) **d**, **f**. Three independent experiments are integrated into each survival curve (n=90). Mean survival time and statistics are presented in Supplementary Data 1.

Supplementary Figure 1

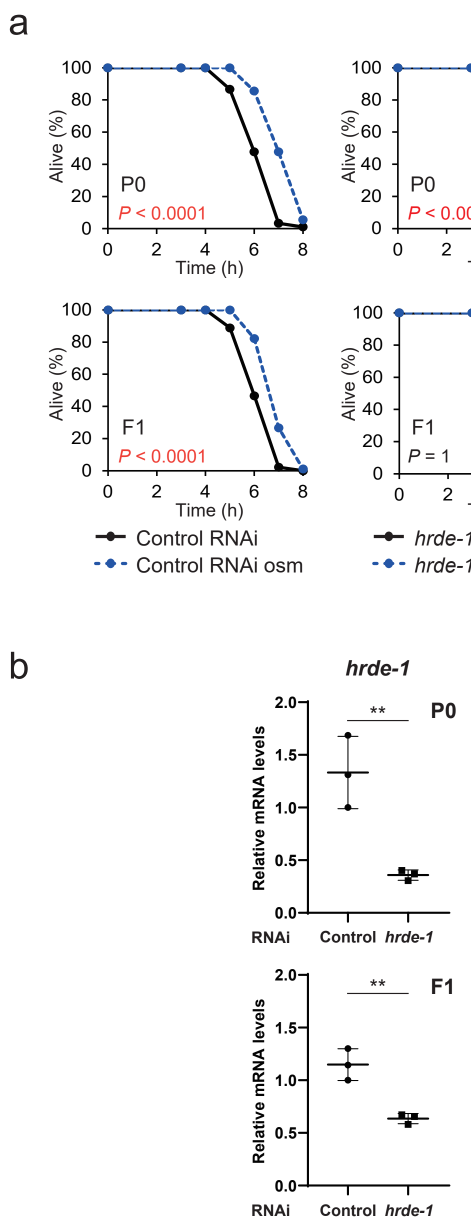

Supplementary Figure 2. Effects of RNAi treatment of *hrde-1* on the stress resistance.

a, Oxidative stress resistance (2.0 mM H_2O_2) of worms treated with RNAi (left, control RNAi; right, *hrde-1* RNAi) in the P0 generation (upper) and the F1 generation (lower). Three independent experiments are integrated into each survival curve (n=90). Mean survival time and statistics are presented in Supplementary Data 1. *P* values were calculated by log-rank test with Bonferroni correction. **b**, Relative mRNA expression levels of *hrde-1* in the P0 generation (upper) and the F1 generation (lower). Error bars represent S.D. of three independent biological replicates. *P* values were calculated by unpaired Student' s *t*-test. $^{**}p<0.01$.

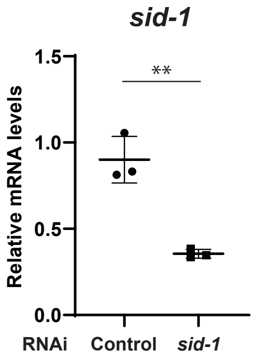

Supplementary Figure 3. Efficiency of *sid-1* RNAi in WT animals.
Relative mRNA expression levels of *sid-1* in WT animals. Error bars represent S.D. of three independent biological replicates. *P* values were calculated by unpaired Student′ s *t* -test. ***p*<0.01.

Supplementary Figure 3

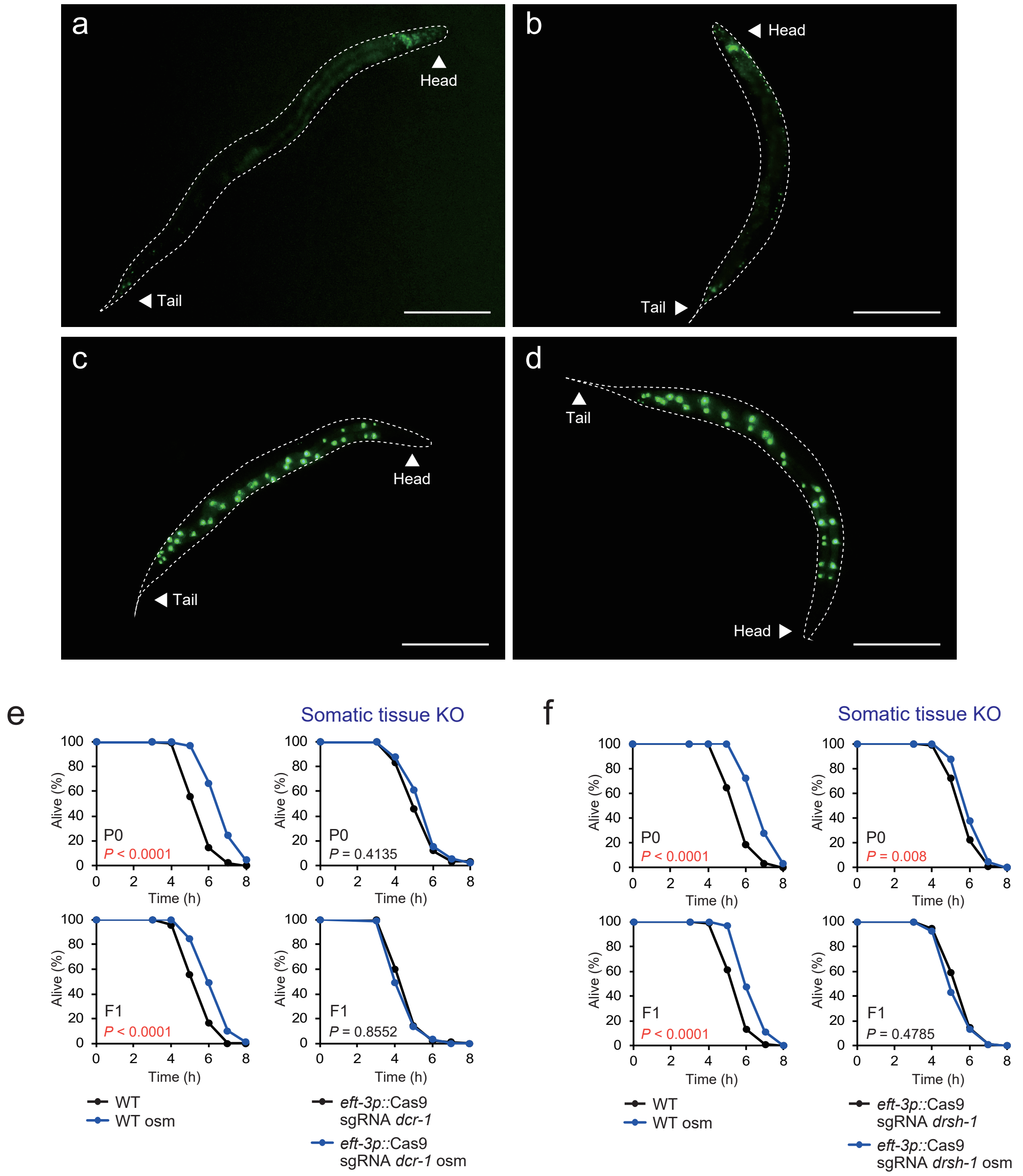

Supplementary Figure 4. Expression pattern of NLS::wGFP and effects of somatic tissue KO of *dcr-1 or drsh-1* on the stress resistance.
a-d, Expression pattern of NLS::wGFP reconstituted from NLS::wGxxFP as observed in neuron-specific *dcr-1* knockout **a**, neuron-specific *drsh-1* knockout **b**, intestine-specific *dcr-1* knockout **c**, and intestine-specific *drsh-1* knockout transgenic worms **d**. Scale bar: 200 µm. **e**, Oxidative stress resistance (1.8 mM H_2O_2) of WT (left) and *dcr-1* knockout in somatic tissues animals (right) in the P0 generation (upper) and the F1 generation (lower). **f**, Oxidative stress resistance of WT (left) and *dcr-1* knockout in somatic tissues animals (right) in the P0 generation (upper) and the F1 generation (lower). Three independent experiments are integrated into each survival curve (n=90). Mean survival time and statistics are presented in Supplementary Data 1. *P* values were calculated by log-rank test with Bonferroni Correction.

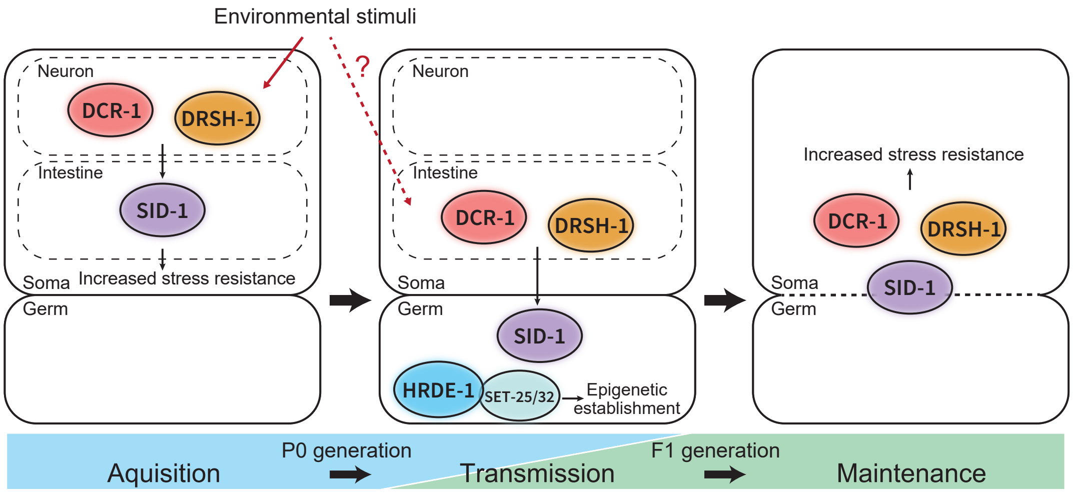

Supplementary Figure 5. Model of this study (See text for detail).

Supplementary Figure 5

Supplementary Table 1. Strains used in study.

Strain	Genotype	Reference
PD8753	*dcr-1(ok247) III / hT2[bli-4(e937)let-?(q782)qIs48] (I; III)*	Grishok et al., 2001
	dcr-1(tm12491)	
WM191	MAGO12 mutant	Gu et al., 2009
VC1138	*drsh-1(ok369) I / hT2[bli-4(e937)let-?(q782)qIs48] (I; III)*	Juang et al., 2013
YY538	*hrde-1(tm1200) III*	Buckley et al., 2012
YY160	*nrde-1(gg88) III*	Buckley et al., 2012
YY156	*nrde-2(gg95) II*	Guang et al., 2010
YY453	*nrde-4(gg129) IV*	Buckley et al., 2012
MT17463	*set-25(n5021) III*	Towbin et al., 2012
VC967	*set-32(ok1457) I*	Ashe et al., 2012
HC196	*sid-1(qt9) V*	Winston et al., 2002
VP303	*rde-1(ne219) V; kbIs7[nhx-2p::rde-1; rol-6(su1006)]*	Espelt et al., 2005
NL2098	*rrf-1(pk1417) I*	Sijen et al., 2001
	set-25(n5021) III;set-32(ok1457) I	This study
	kyEx1901[pCFJ90(myo-2p::mCherry); eft-3p::Cas9; U6p::dcr-1-sgRNA)]	This study
	kyEx1902[pCFJ90(myo-2p::mCherry); rgef-1p::Cas9; U6p::dcr-1-sgRNA)]	This study
	kyEx1903[pCFJ90(myo-2p::mCherry); gly-19p::Cas9; U6p::dcr-1-sgRNA)]	This study
	kyEx1911[pCFJ90(myo-2p::mCherry); eft-3p::Cas9; U6p::drsh-1-sgRNA)]	This study
	kyEx1912[pCFJ90(myo-2p::mCherry); rgef-1p::Cas9; U6p::drsh-1-sgRNA)]	This study
	kyEx1913[pCFJ90(myo-2p::mCherry); gly-19p::Cas9; U6p::drsh-1-sgRNA)]	This study
	kyEx1921[pCFJ90(myo-2p::mCherry); rgef-1p::Cas9; U6p::dcr-1-sgRNA); sur-5p::NLS::wGxxFP dcr-1]	This study
	kyEx1922[pCFJ90(myo-2p::mCherry); gly-19p::Cas9; U6p::dcr-1-sgRNA); sur-5p::NLS::wGxxFP dcr-1]	This study
	kyEx1923[pCFJ90(myo-2p::mCherry); rgef-1p::Cas9; U6p::drsh-1-sgRNA); sur-5p::NLS::wGxxFP drsh-1]	This study
	kyEx1924[pCFJ90(myo-2p::mCherry); gly-19p::Cas9; U6p::drsh-1-sgRNA); sur-5p::NLS::wGxxFP drsh-1]	This study

Supplementary Table 2. Primers used in study.

**RT-qPCR primers**	
act-1	Fwd: 5’-CCCATCAACCATGAAGATCAA-3’
	Rev: 5’-CACATCTGTTGGAAGGTGGA-3’
dcr-1	Fwd: 5’-TTTGGCGAGTTCGGATAGTC-3’
	Rev: 5’-CGAGCTCTTCCCTTTGACTG-3’
drsh-1	Fwd: 5’-GATGTGTCTGATGACTCGAACG-3’
	Rev: 5’-CTTTTACTTCTCCGGTCTTGTCC-3’
hrde-1	Fwd: 5’-TATCCTCGATTGCCGAGAAC-3’
	Rev: 5’-TTCTCCCTTGTCGTCCTTCA-3’
sid-1	Fwd: 5’-AGCAGAAAATTGGCACCATC-3’
	Rev: 5’-CAGCCGTGATCTCTTCAACA-3’
**sgRNA primers**	
dcr-1 sgRNA #1	Fwd: 5’-TGTTGACTGTACGATGGGTTTTAGAGCTAGAAATAGC-3’
	Rev: 5’-TCGTACAGTCAACAATCCAAGAACATCTCGCAATAGGA-3’
dcr-1 sgRNA #2	Fwd: 5’-GCACCATTGGATCAGGGGTTTTAGAGCTAGAAATAGC-3’
	Rev: 5’-TGATCCAATGGTGCAAACAAGAACATCTCGCAATAGGA-3’
drsh-1 sgRNA #1	Fwd: 5’-TTTGAGCATCACAAAGGGTTTTAGAGCTAGAAATAGC-3’
	Rev: 5’-TTGTGATGCTCAAATTGCAAGAACATCTCGCAATAGGA-3’
drsh-1 sgRNA #2	Fwd: 5’-AACAGACATGTAATCGGGTTTTAGAGCTAGAAATAGC-3’
	Rev: 5’-ATTACATGTCTGTTTCTCAAGAACATCTCGCAATAGGA-3’
**wGxxFP primers**	
wGxxFP insert	Fwd: 5’-AATTGGACTAACCCTGATTATTTAAAT-3’
	Rev: 5’-GCCCGTACGGCCGACTAGTA-3’
wGxxFP vector	Fwd: 5’-GTCGGCCGTACGGGCCCTTT-3’
	Rev: 5’-AGGGTTAGTCCAATTTGTGTCCAAGAAT-3’
wGxxFP *dcr-1* #1	Fwd: 5’-CATCGTACAGTCAACAATCCTAACCCTGATTATTTAAAT-3’
	Rev: 5’-GTTGACTGTACGATGGCGGTCCAATTTGTGTCCAAGAAT-3’
wGxxFP *dcr-1* #2	Fwd: 5’-CACCATTGGATCAGGGTGGACTTGTCACTACTTTCTGTT-3’
	Rev: 5’-CCTGATCCAATGGTGCAAATCCAATTTGTGTCCAAGAAT-3’
wGxxFP *drsh-1* #1	Fwd: 5’-CTTTGTGATGCTCAAATTCTAACCCTGATTATTTAAATT-3’
	Rev: 5’-TGAGCATCACAAAGGCGGTCCAATTTGTGTCCAAGAATG-3’
wGxxFP *drsh-1* #2	Fwd: 5’-ACAGACATGTAATCGGAGGCTAACCCTGATTATTTAAAT-3’
	Rev: 5’-CGATTACATGTCTGTTTCTTCCAATTTGTGTCCAAGAAT-3’
**RNAi primers**	
hrde-1 RNAi	Fwd: 5’-AATTGAGCTCAAGATATTCTCCGCGACAAC-3’
	Rev: 5’-AATTGGTACCGCAGGCCAATCGATTCCTCA-3’
