# Supplementary material for: Intertissue small RNA communication mediates the acquisition and inheritance of hormesis in Caenorhabditis elegans
Source: Commun Biol. 2021 Feb 16;4:207. doi: 10.1038/s42003-021-01692-3 (PMC7886853; doi:10.1038/s42003-021-01692-3)
Supplement: Supplementary file 2 — Description of Additional Supplementary Files [file 42003_2021_1692_MOESM2_ESM.pdf]

File name: Supplementary Data 1

Description: Source data for main figures 1, 2, 3, and 4 and Supplementary figures 1, 2, and 4.
